# *DHX37* and *NR5A1* Variants Identified in Patients with 46,XY Partial Gonadal Dysgenesis

**DOI:** 10.3390/life13051093

**Published:** 2023-04-27

**Authors:** Felipe Rodrigues de Oliveira, Taís Nitsch Mazzola, Maricilda Palandi de Mello, Ana Paula Francese-Santos, Sofia Helena V. de Lemos-Marini, Andrea Trevas Maciel-Guerra, Olaf Hiort, Ralf Werner, Gil Guerra-Junior, Helena Fabbri-Scallet

**Affiliations:** 1 Center for Molecular Biology and Genetic Engineering (CBMEG), State University of Campinas (UNICAMP), Campinas 13083-875, Brazil; 2 Postgraduate Program in Child and Adolescent Health, School of Medical Sciences, State University of Campinas (UNICAMP), Campinas 13083-887, Brazil; 3 Center for Investigation in Paediatric, School of Medical Sciences, State University of Campinas (UNICAMP), Campinas 13083-887, Brazil; 4 Interdisciplinary Group for the Study of Sex Determination and Differentiation (GIEDDS), State University of Campinas (UNICAMP), Campinas 13083-887, Brazil; 5 Department of Translational Medicine, School of Medical Sciences, State University of Campinas (UNICAMP), Campinas 13083-887, Brazil; 6 Department of Pediatrics, School of Medical Sciences, State University of Campinas (UNICAMP), Campinas 13083-887, Brazil; 7 Division of Paediatric Endocrinology and Diabetes, Department of Paediatric and Adolescent Medicine, University of Lübeck, 23562 Lübeck, Germany; 8 Institute of Molecular Medicine, University of Lübeck, 23562 Lübeck, Germany

**Keywords:** *DHX37*, *NR5A1*, disorders of sex development, gonadal dysgenesis

## Abstract

The group of disorders known as 46,XY gonadal dysgenesis (GD) is characterized by anomalies in testis determination, including complete and partial GD (PGD) and testicular regression syndrome (TRS). Several genes are known to be involved in sex development pathways, however approximately 50% of all cases remain elusive. Recent studies have identified variants in *DHX37*, a gene encoding a putative RNA helicase essential in ribosome biogenesis and previously associated with neurodevelopmental disorders, as a cause of PGD and TRS. To investigate the potential role of *DHX37* in disorders of sexual development (DSD), 25 individuals with 46,XY DSD were analyzed and putative pathogenic variants were found in four of them. WES analyses were performed on these patients. In *DHX37*, the variant p.(Arg308Gln), recurrent associated with DSD, was identified in one patient; the p.(Leu467Val), predicted to be deleterious, was found together with an *NR5A1* loss-of-function variant in patient 2; and, the p.(Val999Met) was identified in two unrelated patients, one of whom (patient 3) also carried a pathogenic *NR5A1* variant. For both patients carrying *DHX37* and *NR5A1* pathogenic variants, a digenic inheritance is suggested. Our findings support the importance of *DHX37* variants as a cause of disorders of sex development, implying a role in testis development.

## 1. Introduction

Disorders/differences of sex development (DSD) are a group of congenital conditions characterized by alterations in chromosomal, gonadal, or phenotypic features that typically define sex determination and differentiation [1]. The 46,XY subgroup includes individuals presenting undermasculinization due to a lack of androgen synthesis or action, or abnormal testicular differentiation [1]. Although the exact prevalence of this subgroup is unknown, it is estimated to occur in approximately one in 20,000 births [2]. Within this subgroup, anomalies in testis determination or maintenance of testicular tissue during early embryonic development result in 46,XY gonadal dysgenesis (GD), which encompasses complete gonadal dysgenesis (CGD) and partial gonadal dysgenesis (PGD) [1,3].

The complete form of GD consists of individuals with bilateral streak gonads that are typically characterized by wavy ovarian stroma and absence of follicles, normal Müllerian structures, and typical female external genitalia [4]. On the other hand, PGD is characterized by partial testis differentiation, usually a streak gonad on one side of the abdomen and a dysgenetic testis on the other side [4]. In terms of internal genitalia, these individuals typically present Müllerian and Wolffian derivates, and external genitalia with varying degrees of virilization, depending on the amount of functional testicular tissue present in the individual’s gonad [3,5,6].

Testicular regression syndrome (TRS) is a condition that falls within the clinical spectrum of 46,XY GD, which includes subjects with partial or complete disappearance of testicular tissue in one or both gonads during fetal life, after the tissue has been formed [7,8]. It is estimated that TRS affects approximately one in 20,000 boys [8]. Although the exact cause of testicular degeneration remains unclear, it is believed that vascular accidents, such as testis torsion or vascular obstruction due to thrombosis, may explain some cases. However, the clinical diagnosis of TRS is not always straightforward, as remnants of testicular tissue for histopathological analysis may be difficult to find [6,7]. Reports of TRS in consanguineous families, as well as its recurrence in their descendants, suggest that a genetic etiology may also play a role in this condition [9,10].

**Figure 1:**
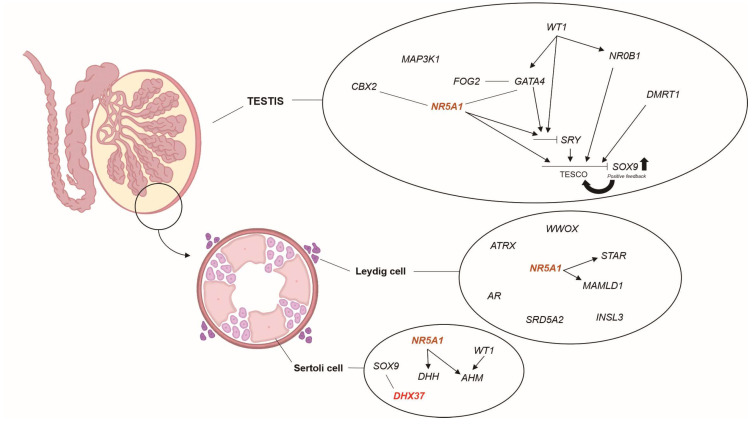
Schematic representation of some of the genes involved in testis determination and differentiation processes, highlighting the *DHX37* (red) and *NR5A1* (orange)*,* described in this study. In addition to *SRY* expression, other genes such as *SOX9*, *GATA4*, *NR5A1*, *AR*, *SRD5A2* and *AMH* are also essential for the male pathway. Genes that promote the action of a downstream target are shown as arrows (Adapted from Maciel-Guerra & Guerra-Júnior [31]).

Several genes are involved in testis determination and differentiation processes (Figure 1), however the genetic etiology of 46,XY GD remains unknown in most of the cases [11]. While variants in *SRY* have been described in approximately 15% of cases and can comprise up to 70% in the CGD subgroup [3,12], their occurrence in PGD is less frequent [13,14]. Through next-generation sequencing (NGS), variants in other genes, including *SOX9*, *DHH*, *GATA4*, *NR0B1*, *FOG2* and *DMRT1*, which are known to play a role in sex determination pathways, have already been identified in individuals with syndromic or non-syndromic forms of 46,XY GD. Nevertheless, only 35 to 40% of all cases have received a definitive genetic diagnosis [15,16].

Pathogenic variants in *NR5A1* are also a prevalent cause of 46,XY GD, defining an additional 11 to 20% of cases [3]. *NR5A1* is an essential transcription factor in regulation of adrenal and gonadal development. It is expressed in both Sertoli and Leydig cells and plays an important role in testis development and function. Initially, variants in this gene were described in patients with a 46,XY karyotype and primary adrenal insufficiency, CGD, and persistence of Müllerian ducts [17,18]. Afterwards, *NR5A1* variants have been linked with a broad spectrum of 46,XY DSD phenotypes, varying from hypospadias and male infertility to testicular dysgenesis, hindering a direct genotype–phenotype correlation [19,20,21,22]. Oligogenic inheritance is also often investigated among those cases, helping to explain several clinical features associated with *NR5A1* variants [23,24,25].

In 2019, heterozygous missense variants in *DHX37* (OMIM *617362) were described in individuals with 46,XY GD or TRS, becoming a new gene associated with 46,XY DSD [11,19,26]. Prior to these findings, only homozygous or compound heterozygous variants in this gene were confirmed to be associated with neurodevelopmental disorders, such as neurodevelopmental disorder with brain anomalies and with or without vertebral or cardiac anomalies (NEDBAVC, OMIM #618731) [27,28].

*DHX37* is an autosomal gene located at chromosomal region 12q24.31 and is composed of 27 exons encoding a putative RNA helicase of 1157 amino acids from the DExD/H-box helicases family. This helicase is divided into four main domains: two of them are RecA-like domains, RecA1 (ATP-binding DEAH-box helicase) and RecA2 (C-terminal helicase), comprising the highly conserved helicase core [26]. The other two domains consist of the helicase-associated 2 domain (HA2) and the oligonucleotide/oligosaccharide-binding fold domain (OB). DHX37 is an RNA-dependent ATPase that plays an essential role in ribosome biogenesis by converting the 21S pre-rRNA into the 18SE pre-rRNA transcript, enabling its subsequent maturation into the 18S rRNA and formation of the small 40S subunit of the ribosome [29,30].

So far, 16 *DHX37* variants have been identified across 43 individuals with different phenotypes of 46,XY DSD [11,19,21,26,32,33] (Supplemental Table S1). Among them, only one case was reported in the homozygous state, suggesting an autosomal dominant inheritance pattern. *DHX37* has been observed to occur at a frequency of 10% to 15% among unresolved cases of GD and of 20% in the TRS group, with a recurrence rate similar to variants in *SRY* and *NR5A1*. *DHX37* is one of the most conserved genes in the human genome and has been found intolerant to loss-of-function and missense variants in the general population [34].

Aiming to disclose *DHX37* variants in a cohort of 46,XY DSD, we report here three heterozygous *DHX37* variants in four 46,XY patients with PGD, with two of them also harboring a pathogenic *NR5A1* variant in their respective genotypes.

## 2. Materials and Methods

### 2.1. Patients Description

In this paper, we describe four unrelated patients with 46,XY PGD in whom *DHX37* and *NR5A1* variants were identified. Initially, a total of 25 patients were analyzed, and among them, 16 were diagnosed with PGD, six with TRS and three with complete GD. A brief review of each patient harboring a pathogenic variant is described below, and their clinical features are summarized in Table 1.

**Patient 1.** At one month of age, Patient 1 was referred to the clinic due to ambiguous external genitalia. The patient had a male sex assignment, with a 1.0 cm phallus, normal male urethral opening without hypospadias, and both gonads palpable in the inguinal canal (Prader 5; EMS 8.0; EGS 8.5). Laboratory data revealed high levels of FSH (12.4 IU/L), and normal levels of LH (2.7 IU/L) and total testosterone (154 ng/dL). Abdominal ultrasound showed no Müllerian derivatives. A diagnosis of 46,XY PGD was established based on bilateral dysgenetic testis revealed from a gonadal biopsy. The patient developed normal puberty at 12 years old but presented with low testosterone levels at 17 years old. Currently, at 21 years old, the patient is using testosterone (1 ampoule IM every 15 days) since the age of 17. The patient’s current Tanner stage is G5P5, with small testes of 6 mL each. The patient has high levels of FSH (34.5 IU/L) and LH (12 IU/L) and presented with azoospermia. He identified his ethnicity as white. The variant c.923G>A/p.(Arg308Gln) was identified in *DHX37* and it was not present in the mother. No DNA from the father was available for analyses.

**Patient 2.** At the age of 17, patient 2 presented with primary amenorrhea, spontaneous pubarche and no thelarche. The patient had female external genitalia with clitoromegaly (3 cm) and no palpable gonads (Prader 2; EMS 0; EGS 1.0). Pelvic ultrasound revealed an absent uterus. Hormonal evaluation indicated high levels of FSH (72.1 IU/L) and LH (13.9 IU/L), and low levels of total testosterone (70 ng/dL). Following gonadectomy, histological analysis revealed dysgenetic testes in both sides with the presence of Sertoli cells, but few Leydig and germ cells, with no ovarian tissue present. Currently, at 23 years old, the patient is undergoing oral estrogenic replacement therapy with T5P5 and has normal adrenal evaluation. She identified her ethnicity as white. Two variants were identified in this patient: the c.1399C>G/p.(Leu467Val) in *DHX37* inherited by the mother, and the c.288_304del/p.(Met98Glyfs*45) in *NR5A1* by the father, who was presumed fertile but was not submitted to clinical examination.

**Patient 3.** Patient 3 was referred to the clinic at the age of 0.5 months due to ambiguous genitalia, with a 2.2 cm phallus length with chordee, a penoscrotal urethral opening, and both gonads palpable in the labioscrotal folds (Prader 4; EMS 6.0; EGS 9.0). After investigation, a male sex assignment was made. Laboratory tests during minipuberty showed high levels of FSH (11.8 IU/L) and LH (7.5 IU/L), with normal values of total testosterone (178 ng/dL). A diagnosis of PGD was confirmed by gonadal biopsy during orchidopexy, which revealed dysgenetic testis bilaterally. Both gonads were preserved, and the patient underwent hypospadias correction at the ages of 2 and 4 years old. Currently, at 9 years old, he is developing normally and has adapted well to the male sex. His adrenal evaluation is normal. His ethnicity was identified as white. This patient also carries two variants in both *DHX37* and *NR5A1* genes, the c.2995G>A/p.(Val999Met) in *DHX37* inherited by the father and the *de novo* c.11C>A/p.(Ser4*) in *NR5A1.*

**Patient 4.** At the age of 12, patient 4 presented at the service with male sex assignment and ambiguous genitalia that had not been previously investigated. The patient had a 4 cm phallus length, a distal penile urethral opening, and both gonads in the labioscrotal folds (Prader 4.0; EMS 8.0; EGS 10.0). Hormonal evaluation indicated high levels of FSH (20.7 IU/L), normal LH (2.8 IU/L) and low levels of total testosterone (20 ng/dL), with G1P2 and both testes with a volume of 2.0 mL. Pelvic ultrasound revealed the absence of Müllerian derivatives. Hypospadias was corrected when the patient was 13 and 15 years old. Currently, at 22 years old, with G5P5, the patient shows azoospermia and both testes with a volume of 5 mL under testosterone replacement every 15 days (1 ampoule IM). He identified his ethnicity as white. The recurrent *DHX37* variant p.(Val999Met) was found in this patient. DNA material from his parents was not available for analysis.

### 2.2. Methods

#### 2.2.1. Sequencing

All patients were analyzed by Sanger sequencing of the entire coding sequence of *DHX37*. Initially, genomic DNA was extracted from peripheral blood leukocytes, according to standard protocols by lysing with proteinase K, extracting with phenol/chloroform, and precipitating with ethanol. All 27 *DHX37* exons including ~150 bp of each of their intron/exon boundaries were amplified by PCR using specific primers designed based on the reference sequence (NM_032656.4/ENST00000308736.7). Independent amplicons were purified in 1% agarose gel electrophoresis with the Wizard SV Gel and PCR clean-up system (Promega, Madison, WI, USA), followed by sequencing with both forward and reverse primers using the Big Dye Terminator v3.1 Cycle Sequencing Kit (ABI PRISM/Life Technologies, Austin, TX, USA). The generated sequences were then analyzed and compared with the *DHX37* reference sequence through the usage of DNA Sequencing Software Chromas Lite v.2.6.6 (Technelysium Pty Ltd., South Brisbane, Australia), and CLC Sequence Viewer 8.0, a free tool included in the QIAGEN CLC Genomics Workbench (QIAGEN Digital Insights, Aarhus, Denmark).

Genomic DNA was extract from saliva (Oragene Kit, OG-500, DNAGENOTEK, Ottawa, ON, Canada) and blood samples obtained from patient 2 and her father, for pyrosequencing analysis. PCR and biotinylated sequencing primers were designed using the PyroMark Assay Design software (Qiagen, Hilden, Germany). The PCR reaction was performed using the Promega Go-Taq Kit (Promega, Walldorf, Germany) and the resulting products were analyzed using the PyroMark Q48 Autoprep instrument (Qiagen, Hilden, Germany).

#### 2.2.2. Whole Exome Sequencing Analysis

Whole Exome Sequencing (WES) was performed for all four patients with *DHX37* variants. Library preparation was carried out according to the manufacturer’s instructions by utilizing Agilent SureSelect Human All ExonV5/V6 capture kit (Agilent Technologies, Santa Clara, CA, USA) and sequenced on a HiSeq2500 System (Illumina, Inc., Sacramento, CA, USA). Burrows-Wheeler Aligner (BWA-MEM, v0.7.17) was used to align the paired end reads to the reference genome. A VCF file containing single nucleotide variants (SNPs), small insertions, and deletions was generated. Variant annotation was performed afterwards using Ensembl Variant Effect Predictor (VEP,v103), while Integrative Genomics Viewer (IGV) was used to evaluate the coverage depth and read quality of the variants, which were filtered to include only those with a minor allele frequency (MAF) < 1% based on GnomAD v2.1.1 Browser and intersected with a DSD gene list, including 150 genes associated with 46,XY DSD phenotypes, based on online databases such as PubMed (https://www.ncbi.nlm.nih.gov/pubmed/ accessed on 2 December 2022), Online Mendelian Inheritance in Man (OMIM, https://www.ncbi.nlm.nih.gov/omim accessed on 2 December 2022), and Human Gene Mutation Database (HGMD, http://www.hgmd.cf.ac.uk/ac/index.php accessed on 2 December 2022).

#### 2.2.3. In Silico Analysis

Online in silico tools were used to determine the potential pathogenicity of the identified nonsynonymous variants on protein function, such as Polyphen-2 (http://genetics.bwh.harvard.edu/pph2/ accessed on 2 December 2022), SIFT (Sorting Intolerant From Tolerant) (http://sift.bii.a-star.edu.sg/ accessed on 2 December 2022), Mutation Taster (http://www.mutationtaster.org/ accessed on 2 December 2022), and Combined Annotation Dependent Depletion (CADD) (https://cadd.gs.washington.edu/snv accessed on 2 December 2022).

**Figure 2:**
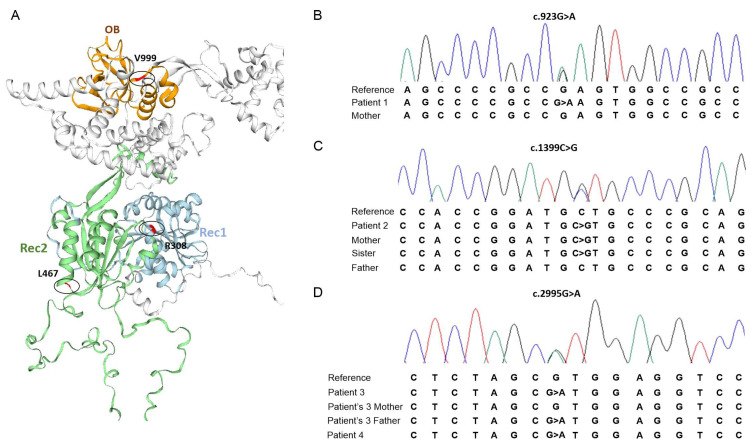
(**A**) Structural model of the DHX37 protein, highlighting the positions (in red) of the variants identified in this study within the conserved domains Rec1 (blue), Rec2 (green) and OB (orange). (**B**) Part of the electropherogram of the c.923G>A/p.(Arg308Gln) variant in patient 1. (**C**) Part of the electropherogram of the c.1399C>G/p.(Leu467Val) variant in patient 2. (**D**) Part of the electropherogram of the c.2995G>A/p.(Val999Met) variant in patients 3 and 4.

Varsome (https://varsome.com/ accessed on 2 December 2022) and the National Center for Biotechnology Information (NCBI) (https://www.ncbi.nlm.nih.gov/ accessed on 2 December 2022) databases were used to verify the variants classification according to the American College of Medical Genetics and Genomics standards and guidelines (ACMG) [35], and their recurrence in the general population, respectively. Structural analyses were performed using PDB ID: 7MQA chain NS as template to generate a homology model using SWISS-MODEL (https://swissmodel.expasy.org/ accessed on 10 January 2023) to observe structural characteristics of the domains where the variants are localized (Figure 2A).

## 3. Results

DNA sequence analyses of *DHX37* in a cohort of 25 46,XY patients with either partial or complete GD or TRS revealed three different variants, each of which was found in heterozygosity in four unrelated patients with PGD (Table 2). 

The first variant identified is a nucleotide exchange from guanine to adenine at position 923 of exon 6, resulting in an amino acid change from arginine to glutamine at position 308 (c.923G>A/p.(Arg308Gln)) identified in patient 1 (Figure 2B). This variant is located within the RecA1 motif of DHX37 helicase protein’s highly conserved core sequence.

The second variant identified in patient 2, is a nucleotide exchange from cytosine to guanine at position 1399 of exon 10, resulting in an amino acid change from leucine to valine at position 467 (c.1399C>G/p.(Leu467Val)), also localized within the protein’s core sequence, in the RecA2 motif (Figure 2C).

The third variant identified in patient 3 and 4 is a nucleotide substitution of guanine by adenine at position 2995 of exon 23, resulting in an amino acid change from valine to methionine at residue 999 (c.2995G>A/p.(Val999Met)). This variant is located within the oligonucleotide/oligosaccharide-binding-like domain (OB) of the DHX37 helicase protein.

WES was performed for all four patients, and in addition to the *DHX37* variants, patients 2 and 3 are also carrier of a heterozygous pathogenic variants in *NR5A1* (Table 2), the c.288_304del/p.(Met98Glyfs*45) and c.11C>A/p.(Ser4*), respectively [20]. By excluding synonymous and intronic variants, considering MAF < 1%, amino acid conservation and position, prediction analysis tools, and literature review, no other relevant variant was identified in DSD-related genes (Supplemental Table S3).

Pyrosequencing analysis was performed on both the saliva and blood samples obtained from the unaffected father of patient 2 for the *NR5A1* variant, and the results indicated the absence of mosaicism in his cells.

As trio analyses is the more appropriate method for delineating the genetic implication of theses variants, Sanger sequencing was performed on the DNA samples of available parents. This analysis clarified the inheritance for both *DHX37* and *NR5A1* variants in patients 2 and 3.

A few other *DHX37* variants, including some rare synonymous variants, were also identified among our cohort. Despite their uncertain significance, we could not exclude that they have a minor effect in the patient’s phenotype. Therefore, these rare variants were reported in Supplemental Table S2.

## 4. Discussion

Although only a few studies have associated *DHX37* variants with a DSD phenotype so far, the high frequency of these variants within different cohorts is notable. Since the first pathogenic variant in this gene was described four years ago as causing DSD, the incidence ratio of *DHX37* variants in 46,XY DSD could be compared with the frequency of *NR5A1* variants among the same group of patients, which is very high. In this study, four out of 25 of the 46,XY patients with GD or TRS carried a pathogenic variant in *DHX37*, indicating a frequency of 16% in our cohort, which is consistent with the results described in the literature [5,6].

It is also intriguing how a helicase, previously associated with a neurodevelopmental disorder, could play a role only in testis development, and this topic has already been the subject of discussion among different sex-determining research groups. McElreavey and colleagues [11] reported the expression of this protein in Sertoli cells and in a subpopulation of interstitial cells, showing also the co-expression of *Sox9* and *Dhx37* in mice. Meanwhile, da Silva et al. [26] found DHX37 expression in Leydig cell cytoplasm and in germ cell lines at different stages of differentiation. Another point raised regarding this specificity is that mothers who carry pathogenic *DHX37* variants have normal ovary development. This fact was also observed in the mother and sister of patient 2, who also carried the c.1399C>G/p.(Leu467Val) *DHX37* variant. According to the authors, the reason for 46,XY specificity could be a transient and incorrect rise in WNT signaling induced by a *DHX37* variant in an XY gonadal cell, which could be sufficient to disturb testis determination, a process that is extremely sensitive to gene dosage [3,6]. In addition, it was suggested that *DHX37* variants associated with DSD may be gain-of-function type, whereas those associated with neurodevelopmental disorder may be loss-of-function variants [6]. Furthermore, the fact that the same variant can lead to either GD phenotype or TRS indicates that this gene is important for both testis determination and maintenance [5].

Among the three main variants described in this study, only the c.923G>A/p.(Arg308Gln) had already been reported in a total of seventeen 46,XY DSD individuals, being the most observed variant in *DHX37* associated to DSD to date (Supplemental Table S1). As for the variant’s pathogenicity, it has been shown that p.(Arg308Gln) weakens the interaction between DNA/RNA at the binding site Ia from the RecA1 domain, consequently diminishing the helicase’s ATPase activity [11]. According to the GnomAD Browser, only one individual carrying this allele was reported in a European population, resulting in a frequency of 0.000065, which is exceptionally rare, corroborating its strong association with DSD.

The first report of the p.(Leu467Val) variant was in a 46,XX girl with neurodevelopmental disorders. This variant was inherited from her unaffected mother and described as compound heterozygous with the p.V731M variant [28], suggesting its pathogenicity. In our study, p.(Leu467Val) was identified in patient 2, inherited from her unaffected mother and described in a heterozygous state. In addition, patient 2 also carried the pathogenic loss-of-function p.(Met98Glyfs*45) variant in *NR5A1,* which could fully explain the patient’s phenotype or have a synergistic effect with the *DHX37* variant. To date, no signs of 46,XY DSD in boys with NEDBAVC syndrome and autosomal recessive inherited *DHX37* variants were reported [6].

The third variant, c.2995G>A/p.(Val999Met), identified in patients 3 and 4, is located within the OB-fold domain. Functional studies in yeast Prp43p DEAH/RHA helicase have shown that RNA binding with the OB-fold is important for the RNA-dependent ATPase activity, and in the presence of RNA, alterations in this domain displayed a reduced ATPase activity of the Prp43p helicase [36]. These results therefore indicate that variants outside the helicase core could also affect its function. Alignment of the sequence region containing the Val999 residue using Clustal Omega software (https://www.ebi.ac.uk/Tools/msa/clustalo/ accessed on 24 March 2023) with five other species, including yeast, revealed that the amino acid Val999 is conserved across all the species (Supplemental Figure S1). Nevertheless, special caution should be given to this variant for two reasons. Firstly, due to its high allele frequency in the African/African-American population, where 54 alleles have been reported, resulting in a frequency of 0.0023, which seems too high to cause a rare condition such as gonadal dysgenesis. Secondly, patient 3 also carries a loss-of-function variant in *NR5A1*, which could fully explain the PGD phenotype, making the p.Val999Met variant a chance finding. Still, we cannot ignore the potential significance of this variant as no in vitro studies have been performed to determine its pathogenicity and possible correlation with DSD.

Another point that needs to be highlighted is the potential for a digenic inheritance in patients 2 and 3, who have pathogenic variants in both *NR5A1* and *DHX37*. Patient 2, in particular, drew our attention since she has the most severe phenotype among the four cases described in this study, with female external genitalia and no palpable gonads. This patient inherited the *DHX37* variant c.1399C>G/p.(Leu467Val) from her unaffected mother and the pathogenic *NR5A1* variant c.288_304del/p.(Met98Glyfs*45) from her clinically healthy father. Due to the absence of phenotype in the father, we considered the possibility of cell mosaicism. For this reason, we performed pyrosequencing for the *NR5A1* variant in two distinct tissues, blood, and saliva. Surprisingly the reference and alternative alleles are equally distributed in both tissues, making a mosaicism unlikely. The inheritance of an *NR5A1* variant by an unaffected father is unusual, and the absence of the phenotype in the father could be attributed to incomplete penetrance in *NR5A1* cases, to his genetic background, or to a different allelic expression pattern. A digenic inheritance could also be a reason for the DSD phenotype in patient 2. We believe that the combination of pathogenic variants in both *NR5A1* and *DHX37* genes could have a synergistic effect on gonadal development and function, leading to a more severe phenotype than expected based on the effects of variants in each gene individually. Individuals carrying *NR5A1* variants and a second putative pathogenic variant, have been previously reported in the literature, and could in fact explain the broad phenotypic spectrum related to this gene in DSD [20,23]. However, only one case of DSD with variants in both *NR5A1* and *DHX37* has been reported, indicating that this combination of variants may be rare but clinically relevant [32]. The possible association between *NR5A1* and *DHX37* in DSD underscores the complex genetic interactions involved in gonadal development and function, however further investigation is needed to fully understand the extent of these interactions.

Since little is known about *DHX37* variants in the literature, we are also reporting some rare synonymous variants identified in our study (Supplemental Table S2). In silico prediction tools usually classify variants as pathogenic or benign based mainly on their impact on protein structure, and nucleotide alterations that do not change the encoded amino acid are initially classified as benign, probably benign or of uncertain significance until further evidence is available. However, a recent study by Shen et al. (2022) demonstrated that synonymous variants can be just as harmful as nonsynonymous variants. By measuring the fitness of thousands of nucleotide alterations inserted in 21 genes of budding yeast *Saccharomyces cerevisiae*, the authors observed that three-quarters of synonymous variants resulted in a significant reduction in fitness. Although the protein sequences remained unaltered, they showed lower expression levels when compared to the wild-type sequence of the yeast. This was due to a reduction of mRNA transcript, leading to a reduced protein coding. The authors highlight that these synonymous variants were associated with altered gene expression, suggesting that they could affect mRNA splicing or stability [37]. The study by Shen et al. provides valuable insights into the functional impact of synonymous variants and emphasizes the importance of a careful consideration of these variants in genomic analyses.

## 5. Conclusions

In summary, our study provides significant evidence supporting the association of *DHX37* variants with DSD phenotypes. We report novel cases and variants that contribute to the currently limited list of described variants. While further investigation is necessary to fully understand the impact of these variants on protein function, existing literature highlights the important role of this gene in sex development.

Our study also emphasizes two other important points. First, the contribution that high-throughput sequencing has made to the field of DSD by enabling the identification and disclosure of new potential candidate genes, such as *DHX37*. Second, the importance of considering oligogenic etiology in cases with variable phenotypes, such as in which *NR5A1* and *DHX37* variants coexist. 

New insights into the complexity of sex development pathways are essential to narrow the wide ratio of elusive diagnosis among DSD cases.

## Figures and Tables

**Table 1:** Clinical and hormonal data from the four patients with variants in *DHX37*.

Clinical and Laboratorial Data	Patient 1	Patient 2	Patient 3	Patient 4
**Sex of rearing**	Male	Female	Male	Male
**Karyotype**	46,XY	46,XY	46,XY	46,XY
**Age at 1st evaluation**	1 month	17 years	0.5 month	12 years
**Diagnosis**	PGD	PGD	PGD	PGD
**Prader**	5	2	4	4
**EMS**	8	0	6	8
**EGS**	8.5	1	9	10
**Uterus**	-	-	-	-
**Gonadal position**	Inguinal/Inguinal	Abdominal/Abdominal	Labioscrotal/Labioscrotal	Labioscrotal/Labioscrotal
**Gonadal histology**	DT/DT	DT/streak	DT/DT	DT/DT
**LH (IU/L)**	2.7	13.9	7.5	2.8
**LH Reference range**	0.6–3.5	0.6–8.5	0.6–3.5	0.6–8.5
**FSH (IU/L)**	12.4	72.1	11.8	20.7
**FSH Reference range**	0.5–4.5	0.5–9.5	0.5–4.5	0.5–9.5
**T (ng/dL)**	154	70	108	20
**T Reference range**	100–300	100–750	100–300	100–750

DT: dysgenetic testes; EGS: external genitalia score; EMS: external masculinization score; FSH: follicle stimulating hormone; PGD: partial gonadal dysgenesis; T: total testosterone; (-): absent.

**Table 2:** *DHX37* and *NR5A1* variants identified in 46,XY PGD patients of this study.

Patient	Gene	Nucleotide/Amino Acid Alteration	SNP ID	Allele Frequency/Allele Count *	In Silico Prediction Tools					Inheritance
					ACMG Guidelines	CADD Score	Mutation Taster	PolyPhen	SIFT	
**1**	***DHX37***	c.923G>A/ p.Arg308Gln	rs1384892917	0.00006490 ^1^/1	Pathogenic	33	Disease causing	Probably damaging	Deleterious	Mother: WT Father: NA
**2**	***DHX37***	c.1399C>G/ p.Leu467Val	rs149331610	0.0002952 ^1^/38	VUS	22.2	Disease causing	Probably damaging	Deleterious	Maternal
	***NR5A1***	c.288_304del/ p.(Met98Glyfs*45)	NA	NA	-	NA	-	-	-	Paternal
**3**	***DHX37***	c.2995G>A/ p.Val999Met	rs148710712	0.002316 ^2^/54	Likely Benign	24.8	Disease causing	Probably damaging	Deleterious	Paternal
	***NR5A1***	c.11C>A/ p.(Ser4*)	NA	NA	-	37	-	-	-	de novo
**4**	***DHX37***	c.2995G>A/ p.Val999Met	rs148710712	0.002316 ^2^/54	Likely Benign	24.8	Disease causing	Probably damaging	Deleterious	NA

NA: not available; SNP ID: single nucleotide polymorphism identification. VUS: variant of unknown significance; WT: wild type. * For this project, data from GnomAD (Genome Aggregation Database) database v2.1.1 were used based on the population with the highest allele frequency: ^1^ European (non-Finnish) population; ^2^ African/African American population.

## Data Availability

No new data were created or analyzed in this study. Data sharing is not applicable to this article.

## Supplementary Materials

The following supporting information can be downloaded at: https://www.mdpi.com/article/10.3390/life13051093/s1, Table S1: *DHX37* variants identified in individuals with 46,XY DSD. Table S2: Recurrent *DHX37* variants identified in 46,XY DSD patients of the study cohort. Table S3: Additional findings obtained by WES after specific filtering. Figure S1: Alignment of the sequence region containing the Val999 residue using Clustal Omega software (https://www.ebi.ac.uk/Tools/msa/clustalo/) with five other species: yeast, mouse, chimpanzee, zebrafish, and pig. The amino acid Val999 is conserved across all the species.
