# Supplementary material for: DHX37 and NR5A1 Variants Identified in Patients with 46,XY Partial Gonadal Dysgenesis
Source: Life (Basel). 2023 Apr 27;13(5):1093. doi: 10.3390/life13051093 (PMC10222664; doi:10.3390/life13051093)
Supplement: Supplementary file 1 [file life-13-01093-s001.zip › Figure S1.pdf]

```
Val999
sp|P53131|PRP43_YEAST            KKRSGAKGYITVKDNQDVLIHPSTVLGH-DAEWVIYNEFVLTSKNYIRTVTSVRP     715
tr|B8JLP0|B8JLP0_DANRE           ----WRNAYKTPLLDDPVFIHPSSALHKTLPEFVVYQEIMETTKMYMRGVCAVQD     993
tr|H0VXV8|H0VXV8_CAVPO           ----WRNAYKTPLLDDPVFIHPSSVLFRELPEFVVYQEIVETTKLYMKGVSAVEE     1005
tr|Q5DTX1|Q5DTX1_MOUSE           ----WKNAYKTPLLDDPVFIHPSSVLFKELPEFVVYQEIVETTKMYMKGVSTVEI     781
sp|Q8IY37|DHX37_HUMAN            ----WRNAYKTPLLDDPVFIHPSSVLFKELPEFVVYQEIVETTKMYMKGVSSVEV     1001
tr|A0A2I3RKE8|A0A2I3RKE8_PANTR   ----WRNAYKTPLLDDPVFIHPSSVLFKELPEFVVYQEIVETTKMYMKGVSSVEV     999
```

**Figure S1.** Alignment of the sequence region containing the Val999 residue using Clustal Omega software (https://www.ebi.ac.uk/Tools/msa/clustalo/) with five other species: yeast, mouse, chimpanzee, zebrafish, and pig. The amino acid Val999 is conserved across all the species.
