# Supplementary material for: DHX37 and NR5A1 Variants Identified in Patients with 46,XY Partial Gonadal Dysgenesis
Source: Life (Basel). 2023 Apr 27;13(5):1093. doi: 10.3390/life13051093 (PMC10222664; doi:10.3390/life13051093)
Supplement: Supplementary file 1 [file life-13-01093-s001.zip › Table S1. Variants described so far in published articles.pdf]

Supplemental Table S1. *DHX37* variants identified in individuals with 46,XY DSD.

DHX37 variant	Genotype	Diagnosis	Inheritance	Reference
c.911C>T / p.Tre304Met	Het	GD	Maternal	McElreavey et al., 2019 [11]
	Het	GD	*De novo*	
	Het	GD	NA	Da Silva et al., 2019 [25]
c.923G>A / p.Arg308Gln	Het	Two brothers with TRS	Paternal	Da Silva et al., 2019 [25]
	Het	TRS	*De novo*	
	Het	TRS	NA	
	Het	Proband with TRS<br>Maternal uncle with TRS	Maternal	
	Het	PGD	*De novo*	
	Het	GD	*De novo*	McElreavey et al., 2019 [11]
	Het	GD	NA	
	Het	GD	NA	
	Het	GD	NA	
	Het	TRS	*De novo*	
	Het	PGD	*De novo*	Buonocore et al., 2019 [18]
	Het	PGD	*De novo*	
	Het	PGD	*De novo*	
	Het	TRS	*De novo*	Zidoune et al., 2021 [32]
	Het	TRS	Maternal	
	Het	TRS	NA	
	Het	PGD	NA	This study
c.1000C>T / p.Arg334Trp	Het	TRS	NA	McElreavey et al., 2019 [11]
	Het	TRS	NA	Zidoune et al., 2021 [32]
c.1001G>T / p.Arg334Leu	Het	GD	*De novo*	McElreavey et al., 2019 [11]
c.1169G>A / p.Arg390His	Het	Idiopathic 46,XY DSD	NA	Zidoune et al., 2021 [32]
c.1399C>G / p.Leu467Val	Het	PGD	Maternal	This study
c.1430C>T / p.Tre477Met	Het	PGD	*De novo*	Buonocore et al., 2019 [18]
	Hom	TRS	Both	Zidoune et al., 2021 [32]
c.1460G>A / p.Arg487His	Het	Idiopathic 46,XY DSD	NA	Zidoune et al., 2021 [32]
c.1474G>C / p.Ala494Pro	Het	Idiopathic 46,XY DSD	NA	Gomes et al., 2022 [5]
c.1784C>T / p.Ser595Phe	Het	Proband with PGD<br>Maternal uncle with TRS	Maternal	Da Silva et al., 2019 [25]
c.1877C>T / p.Ser626Leu	Het	TRS	NA	McElreavey et al., 2019 [11]
c.2021C>T / p.Arg674Trp	Het	Two brothers with TRS	Maternal	Da Silva et al., 2019 [25]
	Het	Two brothers with TRS<br>Maternal uncle with PGD	Maternal	
	Het	TRS	NA	
	Het	PGD	Maternal	
c.2021G>A / p.Arg674Gln	Het	GD	NA	McElreavey et al., 2019 [11]
	Het	GD	*De novo*	
	Het	GD	NA	Globa et al., 2022 [20]
c.2209G>A / p.Ala737Thr	Het	Idiopathic 46,XY DSD	NA	Gomes et al., 2022 [5]
c.2995G>A / p.Val999Met	Het	PGD	Paternal	This study
	Het	PGD	NA	
c.3089G>A / p.Gly1030Glu	Het	TRS	NA	McElreavey et al., 2019 [11]
	Het	TRS	NA	Globa et al., 2022 [20]

CGD: complete gonadal dysgenesis; GD: gonadal dysgenesis; PGD: partial gonadal dysgenesis; TRS: testicular regression syndrome; NA: not available.
