# Supplementary material for: DHX37 and NR5A1 Variants Identified in Patients with 46,XY Partial Gonadal Dysgenesis
Source: Life (Basel). 2023 Apr 27;13(5):1093. doi: 10.3390/life13051093 (PMC10222664; doi:10.3390/life13051093)
Supplement: Supplementary file 1 [file life-13-01093-s001.zip › Table S2. Recurrent variants described in 46,XY DSD patients in our cohort.pdf]

Table S2. Recurrent *DHX37* variants identified among 25 46,XY DSD patients from our cohort.

DHX37 variant	SNP ID	GnomAD frequency	*In silico* prediction tools				
			ACMG Guidelines	CADD score	Mutation taster	PolyPhen-2	SIFT
c.63G>A/ p.Ser61=	rs1434551572	0.000014	Probably benign	5.862	Polymorphism	-	-
c.288G>A/ p.Met96Ile	rs11558556	0.357423	Probably benign	20.6	Polymorphism	Benign	Tolerable
c.1373G>A/ p.Arg458Gln	rs11057939	0.110052	Benign	23.4	Polymorphism	Possibly damaging	Tolerable
c.2025G>A/ p.Thr675=	rs4076777	0.360214	Benign	7.572	Polymorphism	-	-
c.2331A>G/ p.Thr777=	rs10773127	-	Benign	1.720	Polymorphism	-	-
c.2367A>C/ p.Ala789=	rs115567271	0.027030	Benign	0.086	Polymorphism	-	-
c.2499G>T/ p.Arg833=	rs11829165	0.155955	Benign	11.0	Polymorphism	-	-
c.2598G>A/ p.Glu866=	rs4258464	-	Benign	5.911	Polymorphism	-	-
c.2605A>G/ p.Ser869Gly	rs4516060	-	Probably benign	16.6	Polymorphism	Benign	Tolerable
c.2793A>G/ p.Ala931=	rs4429156	-	Probably benign	8.359	Polymorphism	-	-
c.3195C>T/ p.Ala1065=	rs4078216	0.201904	Benign	9.254	Polymorphism	-	-
c.3242G>A/ p.Arg1081Gln	rs4447263	-	Probably benign	0.907	Polymorphism	Benign	Tolerable
