# Supplementary material for: DHX37 and NR5A1 Variants Identified in Patients with 46,XY Partial Gonadal Dysgenesis
Source: Life (Basel). 2023 Apr 27;13(5):1093. doi: 10.3390/life13051093 (PMC10222664; doi:10.3390/life13051093)
Supplement: Supplementary file 1 [file life-13-01093-s001.zip › Table S3. Additional findings obtained by WES after specific filtering.pdf]

Table S3: Additional findings obtained by WES after specific filtering.

Patient	Gene	Nucleotide / Amino acid alteration	Type	Allele frequency / allele count	Zygosity	SNP ID	CADD score	ACMG Guidelines	OMIM
1	WNT4 (NM_030761.5)	c.96G>A / p.Ser32=	synonymous	0.001552 / 55	HET	rs144407094	NA	Likely benign	Mullerian aplasia and hyperandrogenism, AD
2	*MID1 (NM_000381.4)*	c.1014G>A / p.Gly338=	synonymous	0.01123 / 214	HEMI	rs147909430	7.18	Benign	Opitz GBBB syndrome, XLR
3	*FLNA (NM_001110556.2)*	c.4250A>T / p.Tyr1417Phe	missense	NA	HEMI	rs1557177456	13.44	Likely benign	Cardiac valvular dysplasia, X-linked, XL

NA: not available; SNP ID: single nucleotide polymorphism identification; HET: heterozygous; HEMI: hemizygous; AD: autossomal dominant; XLR: X-linked recessive; XL: X-linked.

For this project, data from GnomAD (Genome Aggregation Database) database v2.1.1 was used based on the population with the highest allele frequency.
